# Phase Formation Behavior and Thermoelectric Transport Properties of S-Doped FeSe_2−*x*_S*_x_* Polycrystalline Alloys

**DOI:** 10.3390/mi13122066

**Published:** 2022-11-25

**Authors:** Okmin Park, Se Woong Lee, Sang Jeong Park, Sang-il Kim

**Affiliations:** Department of Materials Science and Engineering, University of Seoul, Seoul 02504, Republic of Korea

**Keywords:** FeSe_2_, FeS_2_, thermoelectric, chalcogenides

## Abstract

Some transition-metal dichalcogenides have been actively studied recently owing to their potential for use as thermoelectric materials due to their superior electronic transport properties. Iron-based chalcogenides, FeTe_2_, FeSe_2_ and FeS_2_, are narrow bandgap (~1 eV) semiconductors that could be considered as cost-effective thermoelectric materials. Herein, the thermoelectric and electrical transport properties FeSe_2_–FeS_2_ system are investigated. A series of polycrystalline samples of the nominal composition of FeSe_2−*x*_S*_x_* (*x* = 0, 0.2, 0.4, 0.6, and 0.8) samples are synthesized by a conventional solid-state reaction. A single orthorhombic phase of FeSe_2_ is successfully synthesized for *x* = 0, 0.2, and 0.4, while secondary phases (Fe_7_S_8_ or FeS_2_) are identified as well for *x* = 0.6 and 0.8. The lattice parameters gradually decrease gradually with S content increase to *x* = 0.6, suggesting that S atoms are successfully substituted at the Se sites in the FeSe_2_ orthorhombic crystal structure. The electrical conductivity increases gradually with the S content, whereas the positive Seebeck coefficient decreases gradually with the S content at 300 K. The maximum power factor of 0.55 mW/mK^2^ at 600 K was seen for *x* = 0.2, which is a 10% increase compared to the pristine FeSe_2_ sample. Interestingly, the total thermal conductivity at 300 K of 7.96 W/mK (*x* = 0) decreases gradually and significantly to 2.58 W/mK for *x* = 0.6 owing to the point-defect phonon scattering by the partial substitution of S atoms at the Se site. As a result, a maximum thermoelectric figure of merit of 0.079 is obtained for the FeSe_1.8_S_0.2_ (*x* = 0.2) sample at 600 K, which is 18% higher than that of the pristine FeSe_2_ sample.

## 1. Introduction

Thermoelectric materials have been widely studied in recent years, owing to their ability to convert waste thermal gradients into electrical energy [1]. The energy-conversion efficiency of thermoelectric materials can be evaluated using the dimensionless thermoelectric figure of merit, *zT*, which is expressed by the following Equation (1).

(1) $$zT=\frac{\sigma S^{2}}{\kappa_{tot}}T$$

where *σ*, *S*, *T*, and *κ_tot_* are the electrical conductivity, Seebeck coefficient, absolute temperature, and total thermal conductivity, respectively [2,3,4]. Generally, *κ_tot_* is divided into two terms:

(2) $$\kappa_{tot}=\kappa_{elec}+\kappa_{latt}$$

where *κ_elec_* and *κ_latt_* are the electrical and lattice thermal conductivities, respectively. *zT* can be improved by increasing the power factor (*σ*∙*S*^2^) or reducing *κ_tot_*. However, the trade-off between *σ* and *S* and the proportionate relationship between *σ* and *κ_elec_* make it difficult to improve *zT*. One strategy to obtain a high *zT* is to reduce *κ_latt_* via point-defect phonon scattering, which can be achieved by doping or using a partial solid solution [5,6]. For example, Asfandiyar et al. investigated the thermoelectric properties of alloyed samples in an SnS–SnSe system, and they reported that the *κ_latt_* of the alloyed samples was lower than that of the nonalloyed samples, and that the *zT* of the alloyed samples was greatly enhanced [7].

Transition-metal dichalcogenides (TMDCs) such as HfSe_2_ [8,9], HfTe_2_ [10], MoSe_2_ [11], and SnSe_2_ [12,13] have attracted significant attention because of their high potential for use as thermoelectric materials. Generally, TMDCs have a large effective mass owing to the presence of local *d*- or *f*-orbital electrons, leading to a large magnitude of *S* [14]. Among these TMDCs, FeSe_2_ and FeS_2_ have been actively investigated as promising thermoelectric materials. FeSe_2_ is a *p*-type semiconductor with a narrow direct bandgap of ~1 eV [15]. FeSe_2_ is known to be thermally and structurally stable, as previous studies have reported [16,17]. It has a high carrier concentration, of 10^18^–10^19^ cm^−3^, and is considered a good candidate for thermoelectric applications [17,18]. FeS_2_ is a semiconductor with a narrow bandgap (~1 eV) and is one of the most abundant sulfides in the Earth’s crust [19]. It is nontoxic, inexpensive, and is considered a promising cost-effective thermoelectric material. By combining the first-principles calculations with the Boltzmann transport theory, Harran et al. predicted that the *zT* of FeS_2_ could reach approximately 0.45 [20,21].

In this study, the electrical and thermoelectric transport properties of a series of polycrystalline FeSe_2−*x*_S*_x_* (*x* = 0, 0.2, 0.4, 0.6, and 0.8) samples of the FeSe_2_–FeS_2_ system are investigated. The S substitution for the Se site was successful up to S content of *x* = 0.6. The *σ* increased gradually with the S content, whereas the positive *S* decreased gradually with the S content at 300 K. The weighted mobility of the samples is calculated and analyzed to understand the electrical transport properties better. With an increase in S content, the *κ_latt_* decreases gradually and significantly.

## 2. Experimental Section

Polycrystalline FeSe_2−*x*_S*_x_* (*x* = 0, 0.2, 0.4, 0.6, and 0.8) samples were synthesized via a conventional solid-state reaction in vacuum-sealed quartz tubes. High-purity raw materials: Fe (99.9%, Kojundo Chemical Laboratory Co., Ltd., Tolya, Japan), Se (99.999%, 5 N Plus), and S (99.995%, Sigma-Aldrich, St. Louis, MO, USA) were weighed stoichiometrically and heated at 833 K for 48 h. The obtained ingots were pulverized into powder using a ball-milling machine (SPEX 8000D, SPEX, Costa Mesa, CA, USA). Each powder sample was densified through spark plasma sintering (SPS-1030, Sumitomo Coal Mining Co. Ltd., Japan) at 803 K for 7 min under a pressure of 75 MPa. The crystalline structures of the sintered samples were identified through X-ray diffraction (XRD, D8 Discover, Bruker, Billerica, MA, USA) using Cu K_α1_ radiation. Energy-dispersive X-ray spectroscopy (EDS) and EDS mapping were measured by secondary electron microscopy (SEM). The thermoelectric transport properties (*σ* and *S*) of the samples were measured using a thermoelectric evaluation system (ZEM-3M8, Advance Riko, Kanagawa prefecture, Japan) in the temperature range of 300–600 K. Hall measurements were conducted in the van der Pauw configuration using a Hall measurement system (HMS-5300, Ecopia, Korea) at 300 K. The thickness of the specimen for Hall measurement were 0.75, 0.73, 0.70 and 0.70 mm for *x* = 0, 0.2, 0.4, and 0.6, respectively, and the applied electric current and magnitude of the magnetic field were 20 mA and 0.553 T, respectively. The *κ_tot_* of each sample was calculated as follows:

(3) $$\kappa_{tot}=\alpha \rho C_{p}$$

where *α*, *ρ*, and C*_p_* are the thermal diffusivity, density, and specific heat capacity, respectively. The *α* of the samples were measured in the range of 300–600 K through laser flash analysis (LFA457, Netzsch, Germany). Theoretical densities (*D_x_*) of FeSe_2_ and FeS_2_ are 7.125 and 4.882 g/cm^3^ (JCPDS #01-079-1892 and JCPDS #00-037-0375) respectively, and the *D_x_* for the alloyed samples were considered to be the average value according to the solid solution ratio; the *D*_x_ values for the samples were 7.13, 6.90, 6.68, 6.45, and 6.23 g/cm^3^ for *x* = 0, 0.2, 0.4, 0.6, and 0.8, respectively. The bulk densities for the samples were measured using the Archimedes method, and the relative densities were obtained from *D_x_* and bulk densities. The calculated relative density values were 99.7, 98.9, 98.3, 98.3, and 97.8% for *x* = 0, 0.2, 0.4, 0.6, and 0.8, respectively. The *C_p_* for the samples was measured using a differential scanning calorimeter (DSC8000, Perkin Elmer, Waltham, MA, USA).

## 3. Results and Discussion

**Figure 1:**
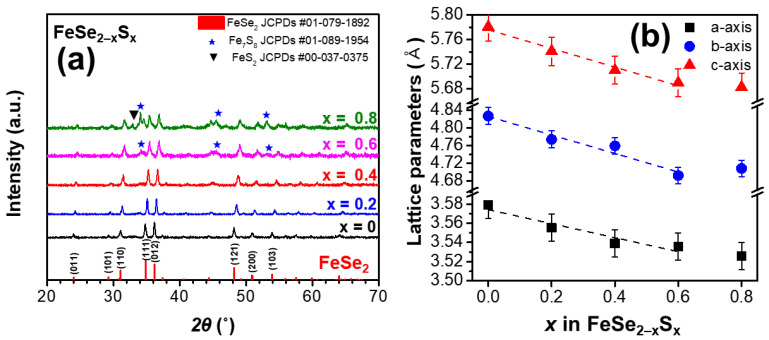
(**a**) XRD patterns of the series of FeSe_2−*x*_S*_x_* (*x* = 0, 0.2, 0.4, 0.6, 0.8, and 1) samples. (**b**) Lattice parameters calculated using the XRD patterns.

Figure 1a shows the XRD patterns of the sintered samples of FeSe_2−*x*_S*_x_* (*x* = 0, 0.2, 0.4, 0.6, and 0.8). The samples with *x* = 0, 0.2, and 0.4 exhibited a single orthorhombic phase (FeSe_2_, JCPDS #01-079-1892) without any impurity. However, for the samples with higher S content (*x* = 0.6 and 0.8), a secondary phase (Fe_7_S_8_, JCPDS #01-089-1954) was identified. For the sample with *x* = 0.8, the peak intensity of the Fe_7_S_8_ secondary phase was increased and a small amount of FeS_2_ was observed. The relative peak intensities remain similar for the samples with *x* = 0, 0.2, 0.4, and 0.6 (See Table S1 in Supplementary Materials). In addition, the grain sizes *D* for the samples (*x* = 0, 0.2, 0.4, and 0.6) were estimated using the Scherrer equation [22]:

(4) $$D=\frac{K\lambda }{\beta \cos\theta }$$

where *K*, *λ*, *θ*, and *β* are the Scherrer constant, the wavelength of the X-ray beam, Bragg angle, and full width at half maximum, respectively. The *K* value of 0.94 was used, assuming the spherical crystallites. The calculated *D* values for the samples were 39.8, 39.2, 32.2, and 24.8 nm for *x* = 0, 0.2, 0.4, and 0.6, respectively. Even though the grain size of *x* = 0.6 is a bit smaller (possibly due to secondary phase formation), it can be known that there is no large difference in microstructures between samples of *x* = 0, 0.2, 0.4, and 0.6. The lattice parameters *a*, *b*, and *c* for the FeSe_2−*x*_S*_x_* (*x* = 0, 0.2, 0.4, 0.6, and 0.8) crystal structures were calculated and are shown with error bars in Figure 1b. All lattice parameters decreased gradually with an increase in S content when *x* < 0.8, which confirmed that S atoms were successfully substituted at Se sites in the FeSe_2_ crystal structure (the ionic radii of S^2−^ and Se^2−^ are 170 and 184 pm, respectively). However, the lattice parameters *a*, *b*, and *c* for *x* = 0.8, exhibited values similar to that for *x* = 0.6, suggesting that further S substitution at Se sites was limited. The EDS-SEM results are shown in Figure S1 and the atomic ratios measured by EDS-SEM are shown in Table S2 (Supplementary Information). The S-excess/Se-deficient regions are seen for *x* = 0.8, where the secondary phases started to be seen. The overall compositional ratios of S increases as S doping increases (Table S2 in Supplementary Information).

**Figure 2:**
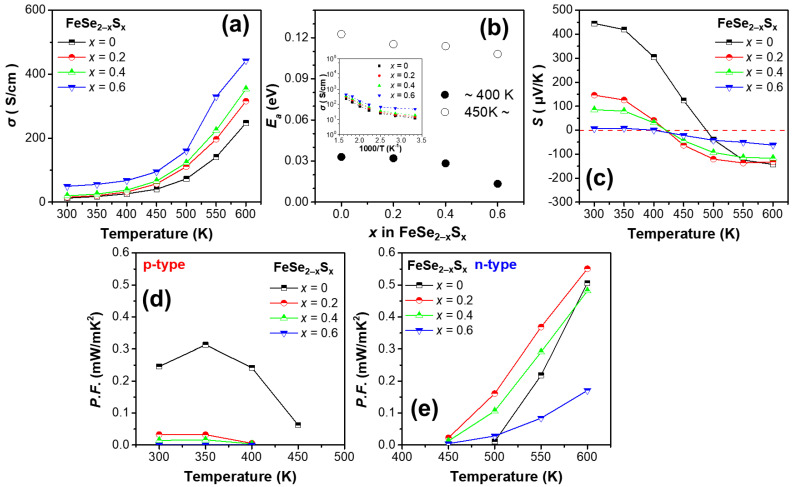
(**a**) *σ* as a function of the temperature for the series of FeSe_2−*x*_S*_x_* (*x* = 0, 0.2, 0.4, 0.6, and 0.8) samples. (**b**) *E_a_* of the samples calculated from *σ* for 300–400 K and 450–600 K. The inset of (**b**) shows logarithmic *σ* as a function of 1000/*T* for the samples. (**c**) *S* as a function of temperature for the samples. Power factors as functions of temperature for the samples in (**d**) *p*- and (**e**) *n*-type regions.

Figure 2a shows the *σ* values of the FeSe_2−*x*_S*_x_* (*x* = 0, 0.2, 0.4, and 0.6) samples. The sample with *x* = 0.8 with extensive secondary phases, which did not show the gradual decrease in lattice parameters, exhibited much higher *σ* values ~440 S/cm at 300 K (Not shown in Figure 2a). The thermoelectric measurement data of the *x* = 0.8 sample is not included due to the non-systematic change due to the existence of the secondary phases. The *σ* of the samples increased with temperature, exhibiting intrinsic semiconducting behavior. The *σ* values were 12.4, 15.3, 19.8, and 49.6 S/cm at 300 K, and 247, 315, 353, 442, and 1170 S/cm at 600 K for *x* = 0, 0.2, 0.4, and 0.6, respectively. The value of *σ* increased gradually with an increase in S content, over the entire temperature range. The relationship between *σ* and *T* can be expressed by the Arrhenius relationship [23]:

(5) $$\sigma =\sigma_{0}\exp\left(-E_{a}/kT\right)$$

where *k* is the Boltzmann constant and *E_a_* is the activation energy. The inset in Figure 2b shows the Arrhenius relationship (logarithmic *σ* as a function of 1000/*T*) for the samples and Figure 2b shows the calculated *E_a_* with respect to *x*. Noticeable changes in the slope were observed at 400–450 K (Inset of Figure 2b). The calculated *E_a_* values of the samples were 0.033, 0.032, 0.028, 0.013, and 0.006 eV in the low-temperature range and 0.123, 0.115, 0.114, 0.108, and 0.048 eV in the high-temperature range for *x* = 0, 0.2, 0.4, 0.6, and 0.8, respectively. The *E_a_* decreased gradually with an increase in S content in both low- and high-temperature ranges.

Figure 2c shows *S* as a function of the temperature for the FeSe_2−*x*_S*_x_* (*x* = 0, 0.2, 0.4, and 0.6) samples. The *S* values of the samples at 300 K were 445, 146, 85, and 6 μV/K for *x* = 0, 0.2, 0.4, and 0.6, respectively. All the samples exhibited positive *S* values at 300 K, showing *p*-type conduction: however, the conduction changes to *n*-type at 400–500 K. At higher temperatures, the *S* values of all the samples became negative and the *S* values of the samples at 600 K were gradually decreases from −143 to −62 μV/K. Interestingly, the magnitude of *S* decreased gradually with increasing S content at both 300 and 600 K.

Figure 2d,e shows the calculated power factors of the samples where the conduction is *p*- and *n*-type, respectively, according to the temperature range. In Figure 2d, the power factor of *p*-type conduction was the highest for the pristine FeSe_2_ sample (*x* = 0), exhibiting a maximum value of 0.31 mW/mK^2^ at 350 K. The power factors of the alloyed samples (*x* = 0, 0.2, 0.4, and 0.6) were lower than that of the pristine sample, despite the increase in *σ*, mainly owing to the significant decrease in *S* at the low-temperature range. However, when considering the power factor of the *n*-type conduction, the power factor at 600 K of *x* = 0.2 and 0.4 was comparable to the pristine sample (the power factors of the samples at 600 K were 0.50, 0.55, 0.48, 0.17, and 0.051 mW/mK^2^ for *x* = 0, 0.2, 0.4, 0.6, and 0.8, respectively). The power factor of the sample with *x* = 0.2 was the highest, which could be attributed to an increase in *σ* and a small decrease in the magnitude of *S*. Therefore, a maximum power factor of 0.55 mW/mK^2^ was achieved for the sample with *x* = 0.2 at 600 K, which was 10% higher than that of pristine FeSe_2_. The sample of *x* = 0.6 exhibits lower power factors due to a large decrease in the magnitude of *S*. 

**Figure 3:**
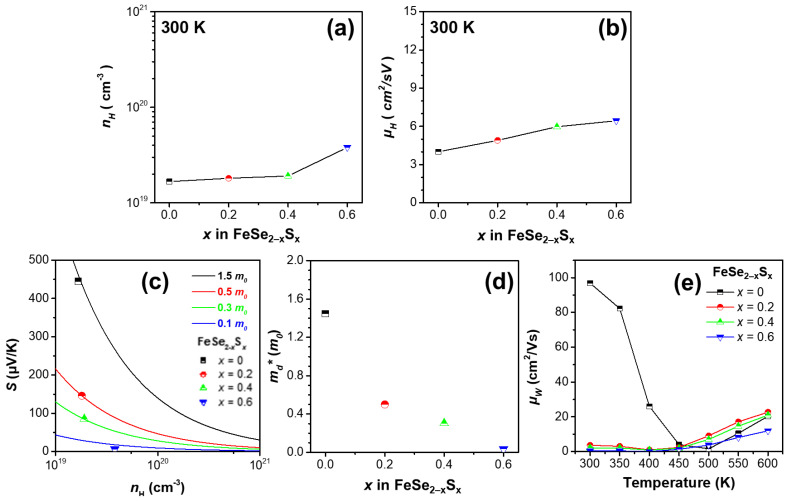
(**a**) *n*_H_ and (**b**) *μ*_H_ of the series of FeSe_2−*x*_S*_x_* (*x* = 0, 0.2, 0.4, and 0.6) samples. (**c**) *S* as a function of *n*_H_ (Pisarenko plot) at 300 K. (**d**) m_d_^*^ as a function of *x* in FeSe_2−*x*_S*_x_* (*x* = 0, 0.2, 0.4, and 0.6). (**e**) *μ_w_* as a function of temperature for the samples.

Figure 3a,b present the Hall carrier concentration (*n*_H_) and Hall mobility (*μ*_H_) of the samples, measured at 300 K. The *n*_H_ values were 1.67 × 10^19^, 1.81 × 10^19^, 1.91 × 10^19^, and 3.80 × 10^19^ cm^−3^ and the *μ*_H_ values were 4.01, 4.90, 5.97, and 6.44 cm^2^/Vs, for *x* = 0, 0.2, 0.4, and 0.6, respectively. All the samples exhibited positive *n*_H_ values at 300 K, and *n*_H_ and *μ*_H_ increased gradually with an increase in *x*. Thus, the increase in the *σ* of the samples could be due to the simultaneous increase in *n*_H_ and *μ*_H_. Furthermore, an increase in carrier concentration generally leads to a decrease in the magnitude of *S*, according to the Mott relationship [24]:

(6) $$S=\frac{8\pi^{2}k^{2}}{3eh^{2}}m_{d}^{*}T{\left(\frac{\pi }{3n}\right)}^{\frac{2}{3}},$$

where m_d_*, *e*, and *h* are the density-of-state effective mass, elementary charge, and Planck’s constant, respectively. Therefore, the decrease in the magnitude of *S* for the alloyed samples at 300 K could be attributed to the increase in *n*_H_. Figure 3c,d show the m_d_* values of the samples at 300 K calculated using the measured *S* and *n*_H_, based on the relationship in Equation (6). The m_d_* values of the samples were 1.45, 0.50, 0.30, and 0.04 *m*_0_ for *x* = 0, 0.2, 0.4, and 0.6, respectively. The m_d_* values decreased gradually with an increase in S content.

Figure 3e shows the weighted mobility (*μ_w_*) of the samples, obtained for a better understanding of the thermoelectric properties of the samples. The *μ_w_* was calculated using the measured *σ* and *S* from a simple analytic form that approximates the exact Drude–Sommerfeld free-electron model for |*S*| > 20 μV/K [25]:

(7) $$\mu_{w}=\frac{3h^{3}\sigma }{8\pi e{\left(2m_{e}kT\right)}^{3/2}}\left[\frac{\exp\left[\frac{\left|S\right|}{k/e}-2\right]}{1+\exp\left[-5\left(\frac{\left|S\right|}{k/e}-1\right)\right]}+\frac{\frac{3}{\pi^{2}}\frac{\left|S\right|}{k/e}}{1+\exp\left[5\left(\frac{\left|S\right|}{k/e}-1\right)\right]}\right],$$

where *m_e_* is the electron mass. *μ_w_* is closely related to the theoretically optimum electrical performance of a thermoelectric material, and is relevant to the maximum power factor when *n*_H_ is tuned properly [26]. Therefore, the *μ_w_* trend was very similar to that of the power factor. The values of *μ_w_* at 600 K were 20.3, 22.7, 20.9, and 11.9 cm^2^/Vs for *x* = 0, 0.2, 0.4, and 0.6, respectively. The *μ_w_* increased initially at *x* = 0.2 and decreased gradually at *x* > 0.2, which was in agreement with the power factor trend at 600 K. 

**Figure 4:**
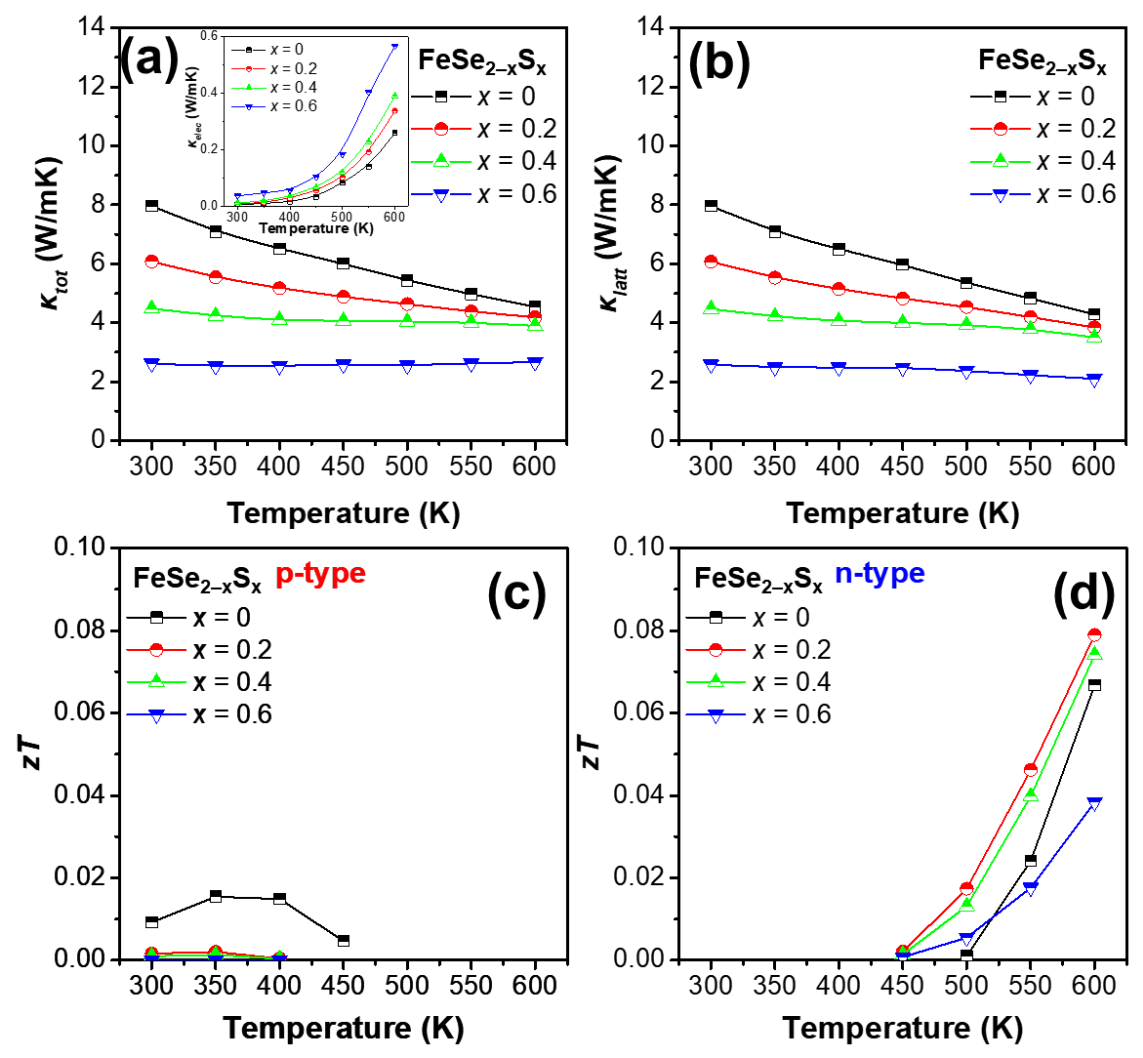
(**a**) *κ_tot_* and (**b**) *κ_latt_* as functions of temperature for the series of FeSe_2−*x*_S*_x_* (*x* = 0, 0.2, 0.4, and 0.6) samples. The inset of (**a**) shows *κ_elec_* as a function of temperature for the samples. *zT* as a function of temperature for the samples in (**c**) *p*- and (**d**) *n*-type regions.

Figure 4a,b show the *κ_tot_* and *κ_latt_* of the samples as functions of temperature. The inset of Figure 4a shows the temperature dependence of *κ_elec_*. The *κ_elec_* of the samples was calculated according to the Wiedemann–Franz law [27]:

(8) $$\kappa_{elec}=L\sigma T$$

where *L* is the Lorenz number. Subsequently, the *κ_latt_* was calculated by subtracting *κ_elec_* from *κ_tot_*. The electronic contribution to *κ_tot_* was not very significant; thus, *κ_tot_* and *κ_latt_* exhibited similar values. The *κ_latt_* values of the samples were 7.96, 6.07, 4.47, and 2.58 W/mK at 300 K and 4.29, 3.85, 3.51, and 2.10 W/mK at 600 K, for *x* = 0, 0.2, 0.4, and 0.6, respectively. The *κ_latt_* decreased gradually as the S content increased, over the entire temperature range, which was attributed to the point-defect phonon scattering caused by the partial substitution of S atoms at Se sites (the atomic masses of S and Se are 32.065 and 78.96 amu and the ionic radii of S^2−^ and Se^2−^ are 170 and 184 pm, respectively).

Figure 4c,d shows the calculated *zT* values of *p*- and *n*-type conduction, respectively. In Figure 4c, the maximum *zT* of the pristine sample was ~0.02, and the alloyed samples exhibited even lower *zT* values below 0.005. On the other hand, in Figure 4d of *n*-type conduction of high-temperature range, the samples with *x* = 0.2 and 0.4 exhibited *zT* values higher than that of the pristine sample, mainly owing to the decrease in *κ_tot_*. Consequently, the FeSe_1.8_S_0.2_ (*x* = 0.2) sample exhibited a maximum *zT* value of 0.079, which was approximately 18% higher than that of the pristine FeSe_2_ sample. 

## 4. Conclusions

A series of FeSe_2−*x*_S*_x_* (*x* = 0, 0.2, 0.4, 0.6, and 0.8) polycrystalline samples were synthesized by a conventional solid-state reaction, and their thermoelectric transport properties were examined in an effort to search for the cost-effective thermoelectric materials. A single orthorhombic FeSe_2_ phase was successfully synthesized for *x* = 0, 0.2, and 0.4; however, a secondary phase (Fe_7_S_8_ or FeS_2_) was identified for *x* = 0.6 and 0.8. The lattice parameters decreased gradually with an increase in S content for *x* < 0.8, suggesting that S atoms were substituted at the Se sites in the FeSe_2_ crystal structure. The electrical conductivity increased gradually with an increase in S content, whereas the magnitude of *S* decreased gradually with an increase in S content. As a result, the sample with *x* = 0.2 exhibited a maximum power factor of 0.55 mW/mK^2^ at 600 K. The total thermal conductivity decreased significantly with an increase in S content, and thus a maximum thermoelectric figure of merit value of 0.079 was obtained for the FeSe_1.8_S_0.2_ (*x* = 0.2) sample at 600 K, which was approximately 18% higher than that of the FeSe_2_ (*x* = 0) sample.

## Data Availability

Data available upon reasonable request.

## Supplementary Materials

The following supporting information can be downloaded at: https://www.mdpi.com/article/10.3390/mi13122066/s1, Figure S1: EDS-SEM results for FeSe_2−*x*_S*_x_* (*x* = 0, 0.2, 0.4, 0.6 and 0.8); Figure S2: (a) *σ*, (b) *S*, and (c) *PF* as a function of temperature for the FeSe_2_ sample, measured after ~180 days from the initial measurement for cycling test; Figure S3: Estimated *E_g_* for the series of FeSe_2−*x*_S*_x_* (*x* = 0, 0.2, 0.4 and 0.6) samples using Goldsmid-Sharp empirical formular; Table S1: The relative peak intensities for (111), (012), (121), (011), (200) and (103) planes for FeSe_2−*x*_S*_x_* (*x* = 0, 0.2, 0.4, and 0.6) in the X-ray diffraction data; Table S2: Atomic ratios measured by EDS-SEM for FeSe_2−*x*_S*_x_* (*x* = 0, 0.2, 0.4, 0.6 and 0.8). References [16,28] are cited in the supplementary materials.
