# Supplementary material for: Phase Formation Behavior and Thermoelectric Transport Properties of S-Doped FeSe2−xSx Polycrystalline Alloys
Source: Micromachines (Basel). 2022 Nov 25;13(12):2066. doi: 10.3390/mi13122066 (PMC9784414; doi:10.3390/mi13122066)
Supplement: Supplementary file 1 [file micromachines-13-02066-s001.zip › micromachines-1999540-supplementary.pdf]

Supplementary Information

Phase Formation Behavior and Thermoelectric Transport Properties of S-Doped $FeSe_{2-x}S_x$ Polycrystalline Alloys

Okmin Park †, Se Woong Lee †, Sang Jeong Park and Sang-il Kim *

Department of Materials Science and Engineering, University of Seoul, Seoul 02504, Republic of Korea; zcsd1523@uos.ac.kr (O.P.); lswprawn245@uos.ac.kr (S.W.L.); psj1213@uos.ac.kr (S.J.P.)
* Correspondence: sang1.kim@uos.ac.kr
† These authors contributed equally to this work.

S1. Relative peak intensities in X-ray diffraction

Table S1. The relative peak intensities for (111), (012), (121), (011), (200) and (103) planes for $FeSe_{2-x}S_x$ (x = 0, 0.2, 0.4, and 0.6) in the X-ray diffraction data.

x	Relative intensities for (hkl) planes (%)					
	(111)	(012)	(121)	(011)	(200)	(103)
0	100	115	77.0	28.1	36.0	38.1
0.2	100	99.9	62.7	21.5	28.0	30.0
0.4	100	102	63.5	22.6	28.6	33.3
0.6	100	103	76.4	25.3	39.1	32.7

S2. Energy-dispersive X-ray spectroscopy by scanning electronic microscope

Energy-dispersive X-ray spectroscopy (EDS) and EDS mapping were measured by secondary electron microscopy (SEM). The EDS-SEM results are shown in Figure S1 and the atomic ratios measured by EDS-SEM are shown in Table S2. The S-exess/Se-deficient regions are seen for x = 0.8, where the secondary phases started to be seen. The overall compositional ratios of S increases as S doping increases.

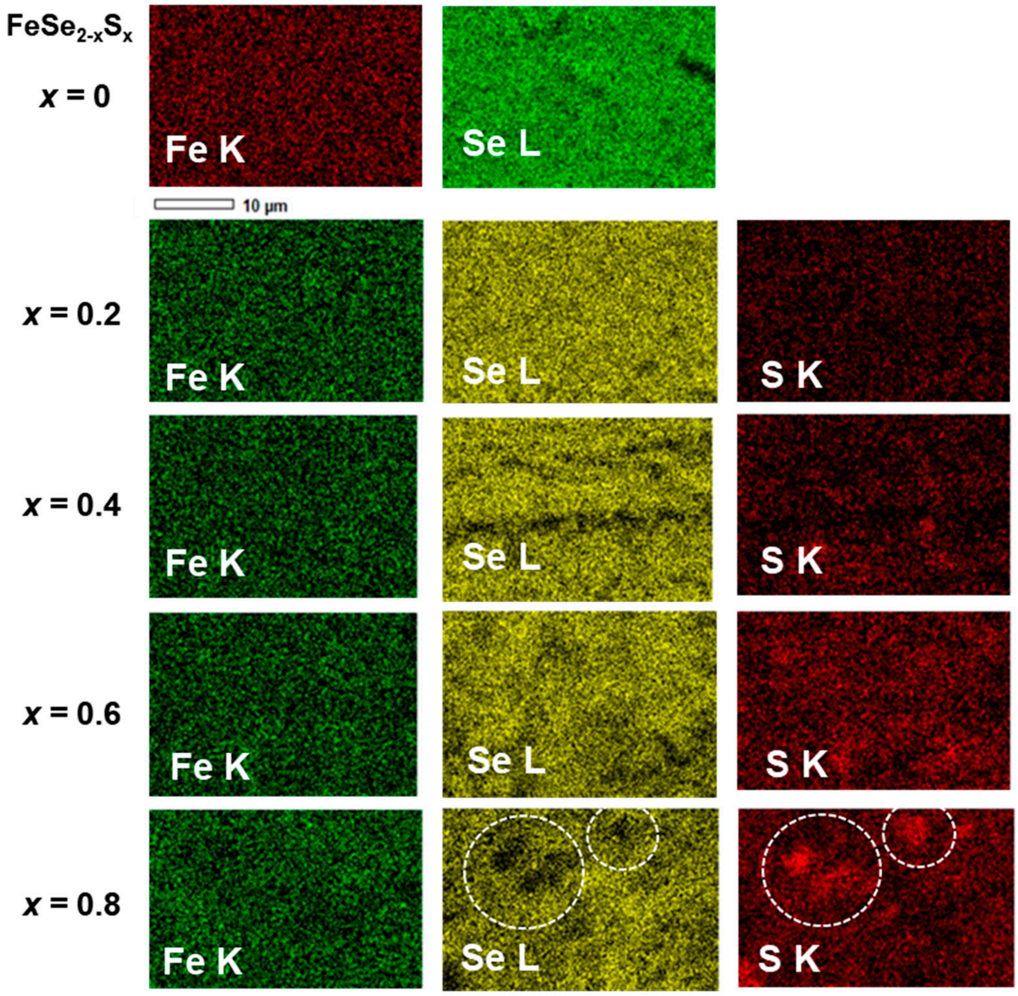

Figure S1. EDS-SEM results for $FeSe_{2-x}S_x$ (x = 0, 0.2, 0.4, 0.6 and 0.8). From x = 0.8, S excess/Se deficient regions are seen.

Table S2. Atomic ratios measured by EDS-SEM for $FeSe_{2-x}S_x$ (x = 0, 0.2, 0.4, 0.6 and 0.8).

$FeSe_{2-x}S_x$	x = 0	x = 0.2	x = 0.4	x = 0.6	x = 0.8
Fe	0.34	0.33	0.34	0.33	0.38
Se	0.66	0.61	0.54	0.48	0.34
S	-	0.06	0.12	0.19	0.28

S3. Thermal cycling effect

To investigate the effect of thermal cycling on the thermoelectric properties of $FeSe_2$, the electrical conductivity σ and Seebeck coefficient S for $FeSe_2$ sample were measured second time after ~ 180 days from the initial measurement for cycle test using a thermoelectric evaluation system (ZEM-3M8, Advance Riko, Japan) in the temperature range of 300–600 K. Figure S1(a) and S1(b) show the measured σ and S for the $FeSe_2$ sample second times with the results of the initial measurement, respectively. As a result, the first and second measurements exhibited the almost same values, suggesting that the thermal cycling effect on the thermoelectric properties of $FeSe_2$ is marginal. This result is in agreement with the previous report [1]. Figure S1(d) shows the *PF* values for the second measurement, and it is almost identical to the initial measurement within error range.

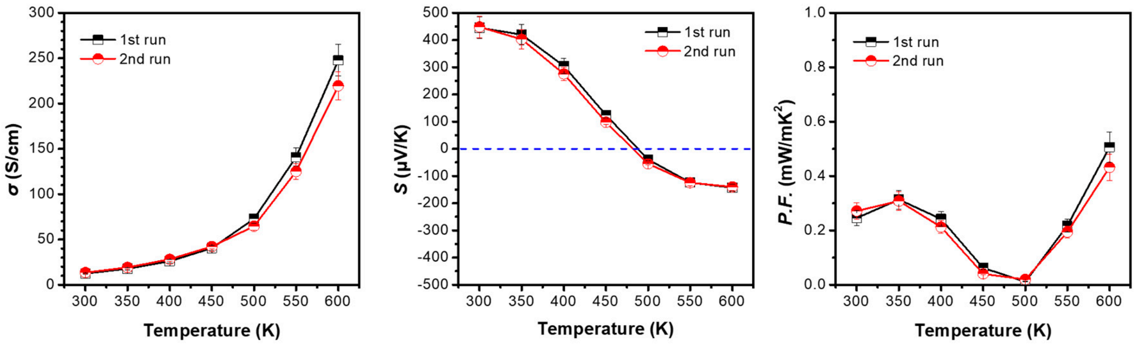

Figure S2. (a) σ, (b) S, and (c) PF as a function of temperature for the $FeSe_2$ sample, measured after ~ 180 days from the initial measurement for cycling test.

S4. Band gap estimation

The band gap E_g as a function of x for the series of $FeSe_{2-x}S_x$ (x = 0, 0.2, 0.4, and 0.6) samples were estimated from the maximum magnitude of the Seebeck coefficient, $|S|_{max}$ and the corresponding temperature T_{max}, using the Goldsmid-Sharp empirical equation [2]:

$$E_g = 2e|S|_{max}T_{max}, \quad \text{(S1)}$$

where e is the elementary charge. The estimated E_g are shown in Figure S2, and the E_g values for the samples were 0.267, 0.088, 0.140, and 0.075 eV for x = 0, 0.2, 0.4, and 0.6, respectively. The E_g for the alloyed samples (x = 0.2, 0.4, and 0.6) were considerably reduced compared to that for the $FeSe_2$ sample, suggesting that substitution of S atoms at Se sites can facilitate electron carrier excitation. This result is in accordance with the fact that Hall carrier concentration and σ for the alloyed samples were enhanced compared to those for the $FeSe_2$ sample.

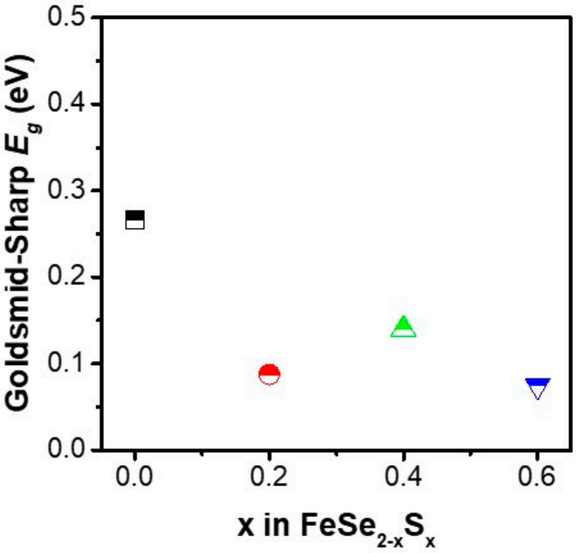

Figure S3. Estimated E_g for the series of $FeSe_{2-x}S_x$ (x = 0, 0.2, 0.4 and 0.6) samples using Goldsmid-Sharp empirical formular.

References

[1] Li, G.; Zhang, B.; Rao, J.; Herranz Gonzalez, D.; Blake, G.R.; de Groot, R.A.; Palstra, T.T. Effect of vacancies on magnetism, electrical transport, and thermoelectric performance of marcasite $FeSe_{2-\delta}$ ($\delta = 0.05$). *Chem. Mat.* **2015**, *27*, 8220.

[2] Goldsmid, H.J.; Sharp, J.W. Estimation of the thermal band gap of a semiconductor from seebeck measurements. *J. Electron. Mater.* **1999**, *28*, 869.
